# Whole brain radiation therapy plus focal boost may be a suitable strategy for brain metastases in SCLC patients: a multi-center study

**DOI:** 10.1186/s13014-020-01509-3

**Published:** 2020-03-25

**Authors:** Meng Ni, Aijun Jiang, Wenju Liu, Yanxing Sheng, Haiyan Zeng, Ning Liu, Qingxiao Gao, Yong Wang, Jinming Yu, Shuanghu Yuan

**Affiliations:** 1 Cheeloo College of Medicine, Shandong University, Jinan, Shandong China; 2 Department of Radiation Oncology, Shandong Cancer Hospital and Institute, Shandong First Medical University and Shandong Academy of Medical Sciences, 440 Jiyan Road, Jinan, 250117 Shandong China; 3 Department of Radiation Oncology, The Affilated Hospatial of Xuzhou Medical University, Xuzhou, Jiangsu China; 4 Department of Radiation Oncology, Liaocheng People’s Hospital, Liaocheng, Shandong China; 5 Binzhou Medical University Hospital, Binzhou, Shandong China; 6 Shandong Cancer Hospital and Institute-Shandong Cancer Hospital Affiliated to Shandong University, Jinan, Shandong China

**Keywords:** SCLC, Brain metastases, WBRT, SRS, WBRT plus focal radiation boost

## Abstract

**Background:**

The treatment for brain metastases in small cell lung cancer (SCLC) is still controversial. The purpose of this study was to compare different brain radiotherapy treatments on SCLC patients with brain metastases.

**Methods:**

In this multi-center retrospective study, SCLC patients who had undergone whole brain radiation therapy (WBRT) or stereotactic radiosurgery (SRS) for brain metastases from January 2012 to December 2018 were retrospectively screened.

**Results:**

A total of 263 eligible SCLC patients were included in this study, among whom, 73 were women and 190 were men. According to accepted brain radiotherapy, the remaining patients were divided into WBRT plus focal radiation boost (WBRT+boost), WBRT, and SRS groups. In pairwise comparisons of the overall survival (OS), WBRT+boost group led to longer survival than did WBRT both in all patients (17.9 vs 8.7 months; *P* < 0.001) and 140 matched patients (17.9 vs 11.7 months; *P* = 0.045). There were no significant differences in OS between WBRT+boost and SRS groups in all patients (17.9 vs 14.5 months; *P* = 0.432). Among 74 matched patients between WBRT+boost and SRS groups, however, patients who received WBRT+boost led to a longer survival than did SRS alone (21.8 vs 12.9 months; *P* = 0.040). In pairwise comparison of the intracranial progression-free survival time (iPFS), WBRT+boost group also showed survival advantages over WBRT (10.8 vs 6.5 months; *P* = 0.005) and SRS groups (10.8 vs 7.5 months; *P* = 0.032).

**Conclusion:**

Due to the SCLC-derived multiple brain metastases and better survival time, focal radiation boost combined with adjuvant WBRT may be a preferred strategy for SCLC patients with brain metastases.

## Introduction

Brain metastases are the most common central nervous system tumors, and the most common primary site is lung cancer [1]. Among them, the most common pathological type of brain metastases is small cell lung cancer (SCLC) [2, 3]. Due to advanced diagnostic imaging technology and effective treatment, the incidence of brain metastases in SCLC has increased correspondingly [4, 5]. About 10–21% of SCLC patients are diagnosed with brain metastases initially, and 50–80% will develop brain metastases during the course of the disease [6–9]. In addition, SCLC patients are often accompanied by multiple brain metastases and poor prognosis, with the median overall survival (OS) of 3 months (from the diagnosis of brain metastases to death) [2, 3]. According to the NCCN Guidelines for Small Cell Lung Cancer Version 2.2020, whole brain radiotherapy (WBRT) is recommended as a standard treatment for brain metastases in patients with SCLC [10]. However, the optimal strategy for brain metastases in SCLC patients still remains controversial. Previous randomized clinical trials suggested that stereotactic radiosurgery (SRS) can be used for 1–3 brain metastases [11], and even for 5–10 brain metastases [12]. Randomized controlled trial is also conducted to investigate the efficacy of SRS alone in SCLC patients with 1–10 brain metastases [13]. Some studies suggested that WBRT plus a radiation boost increased the survival benefit for brain metastases in a specific situation [14–16]. The purpose is to compare three different radiotherapy methods currently used clinically for brain metastases in SCLC patients in this study.

## Methods

This multicenter study included patients from three medical institutions in China.

**Fig. 1:**
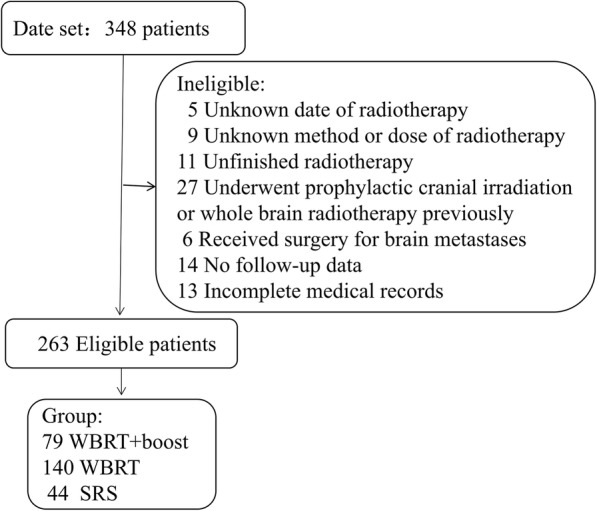
Patients screened and determined to be, eligible or ineligible for inclusion in the study. Abbreviations: WBRT+boost, WBRT plus focal radiation boost

SCLC patients who had undergone WBRT or SRS for brain metastases were retrospectively retrieved from January 2012 to December 2018. Eligible criteria included patients who had pathologically-proven SCLC and imaging-proven brain metastases. In addition, all patients underwent brain radiotherapy. Those patients who underwent prophylactic cranial irradiation (PCI) or WBRT previously were excluded. Those patients who had incomplete medical records at diagnosis or treatment were also excluded. The screening process of patients was listed in Fig. 1. The general characteristics of the patients were recorded, including age, gender, KPS score, number and maximum size of brain metastasis, systemic treatment, type of radiotherapy, and extracranial metastases.

### Treatment

All patients underwent brain radiotherapy after diagnosis of brain metastases. Of these, 79 patients received WBRT plus focal radiation boost (WBRT+boost), 140 patients received WBRT, and 44 patients received SRS. WBRT or focal boost was performed using 3D conformal radiotherapy (3D-CRT) or intensity-modulated radiation therapy (IMRT), and was administered using linear accelerator (Varian Medical Systems). In WBRT, the clinical target volume (CTV) was contoured as the region of the whole brain, with a total dose of 25–45 Gy (2–3 Gy per fraction administered in 10–15 fractions). An extension of 3 mm on CTV was defined as planning tumor volume (PTV). In additional focal boost, the gross tumor volume (GTV) was contoured as contrast-enhanced MRI, with a total dose of 10–20 Gy (2–3 Gy per fraction administered in 5–10 fractions). An expansion of the GTV by 2–3 mm was used as the PTV. SRS was administered using the gamma knife, with a 40–60% isodose line. According to location of the brain metastases, prescribed radiation dose was 10.5–20.5 Gy in 1–3 fractions with 8.5–18.0 Gy per fraction. Due to the condition of patients and the experience of the clinician, the prescribed dose in our study was lower than previously set by the RTOG [17].

According to the condition of patients and the clinical experience of the physician, 80 patients did not receive systemic chemotherapy, and 183 patients received more than 2 cycles of chemotherapy.

### Statistical method

The primary endpoint was OS (death or final follow-up), and the secondary study endpoint was intracranial progression-free survival time (iPFS, the progression of intracranial metastases at the first time or death or final review). IPFS was defined as local failure or the appearance of new metastases. Local failure was assessed by the RECIST version 1.1 criteria [18]. Time to overall survival or intracranial progression-free survival was calculated by Kaplan–Meier method. The 1: 1 optimal propensity score matching method was used between WBRT+boost (treatment) and WBRT (control) or SRS (control) groups to control confounding factors of patients, and the purpose of the matching was to reduce bias in the estimation of treatment effect [19]. Propensity score estimation was calculated by logistic regression analysis. Covariates (ie, extracranial metastases, age, KPS score, symptoms, gender, and maximum size and number of brain metastases) were used to calculate propensity scores. The general characteristics of the patients were calculated by χ2 test before or after matching. Prognostic factors were performed using univariate or multivariate analyses, which was based on Cox models (done at the level of α = 0.05 and forward stepwise likelihood ratio method) [20]. *P* < 0.05 (two-sided) was considered statistically significant in all analyses. All analyses were performed by IBM SPSS 22.0 (IBM Corp).

## Results

### Patient characteristics

A total of 263 eligible patients were included in this study (Fig. 1). Among of them, 185 (70.3%) patients died, 42 (16.0%) patients were lost to follow-up, and 36 (13.7%) patients survived. Of 263 patients (median age, 61 years), 73 (27.8%) were women and 190 (72.2%) were men. The general characteristics of the all patients were listed in Table 1 and were not balanced between the groups.

**Table 1:** Baseline characteristics of patients before propensity score matching

	WBRT + boost	WBRT Alone	SRS Alone	*P* Value
	(*n* = 79)	(*n* = 140)	(*n* = 44)	
Age, No. (%)				**0.563**
< 60	36	67	17	
≥60	43	73	27	
Sex, No. (%)				0.819
Male	55	103	32	
Female	24	37	12	
KPS, No. (%)				0.931
≤80	47	84	25	
> 80	32	56	19	
Number of BMs, No. (%)				< 0.001
1–3	51	34	39	
> 3	28	106	5	
Maximum size of BM, No. (%)				0.170
< 20 mm	36	81	26	
≧20 mm	43	59	18	
Extracranial metastases, No. (%)				0.009
Yes	17	53	6	
None	14	20	6	
Unknown	48	67	32	
Symptoms of BM, No. (%)				0.123
Yes	37	69	14	
None	42	71	30	
Systematic treatment				0.833
CTx	55	99	29	
No-CTx	24	41	15	

Abbreviations: *WBRT+boost* whole brain radiotherapy plus focal radiation boost, *WBRT* whole brain radiation therapy, *SRS* stereotactic radiosurgery, *KPS* Karnofsky Performance Status, *BM* brain metastasis, *CTx* chemotherapy, *No-CTx* no chemotherapy

### Analyses of overall survival time in all patients before propensity score matching

**Fig. 2:**
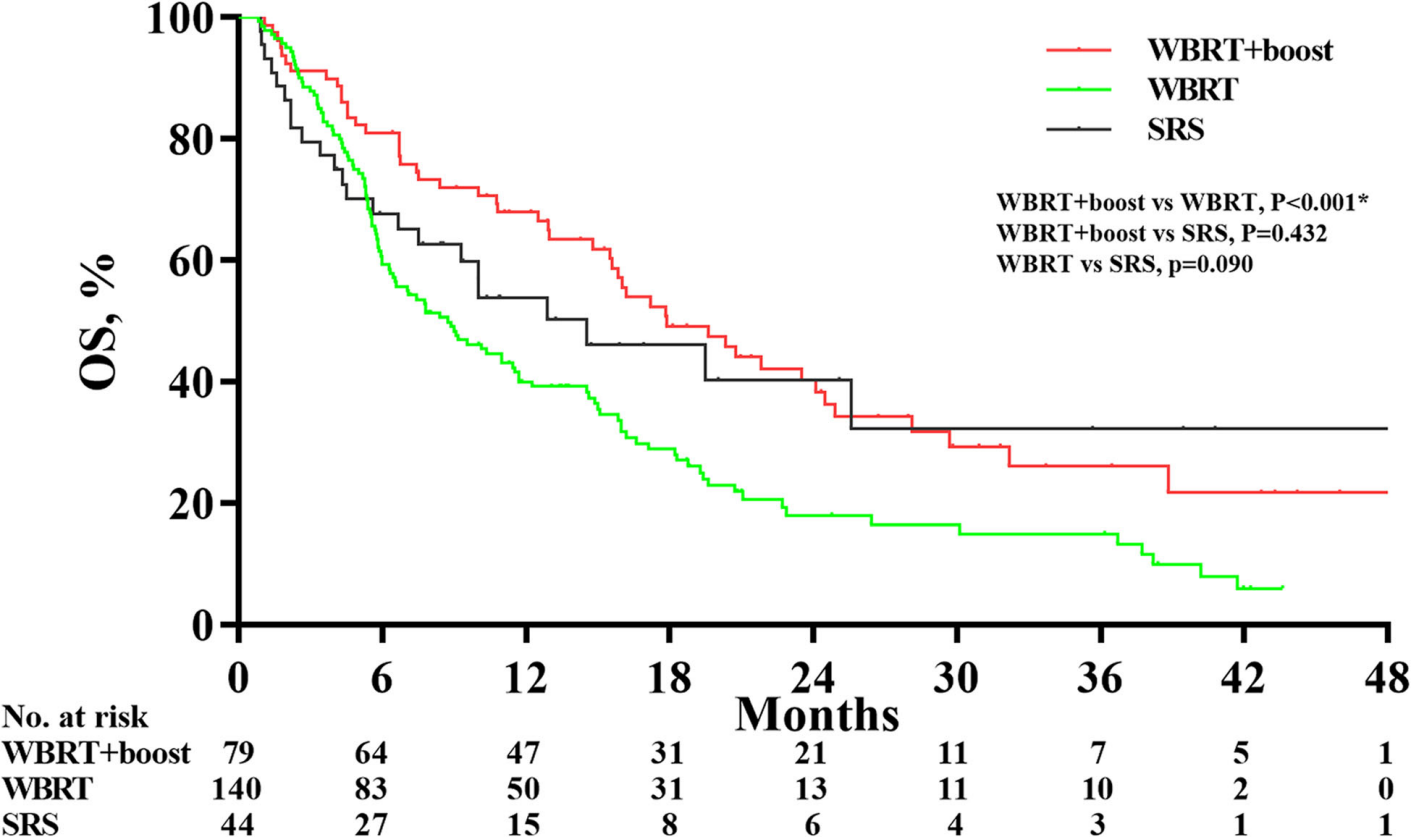
Overall survival analyses according to treatment group in all patients before propensity score matching. The median overall survival (OS) was 17.9 months for patients receiving whole-brain radiation therapy plus focal radiation boost (WBRT+boost), 8.7 months for patients receiving whole-brain radiation therapy (WBRT) alone, and 14.5 months for patients receiving stereotactic radiosurgery (SRS) alone. WBRT+boost group led to a longer OS over WBRT alone group (HR, 1.82; 95% CI, 1.30–2.55; *P* < 0.001)

The final follow-up date was December 30, 2019, with the median follow-up time was 10.1 months. There were significant differences in OS time between WBRT+boost, WBRT, and SRS groups (17.9 vs 8.7 vs 14.5 months; *P* = 0.001). In pairwise comparison of the OS, patients in WBRT+boost group had significantly longer survival than did WBRT (17.9 vs 8.7 months; *P* < 0.001). However, there were no significant differences in OS between WBRT+boost and SRS groups (17.9 vs 14.5 months; *P* = 0.432) or WBRT and SRS groups (8.7 vs 14.5 months; *P* = 0.090) (Fig. 2).

### Analyses of overall survival time after propensity score matching

**Fig. 3:**
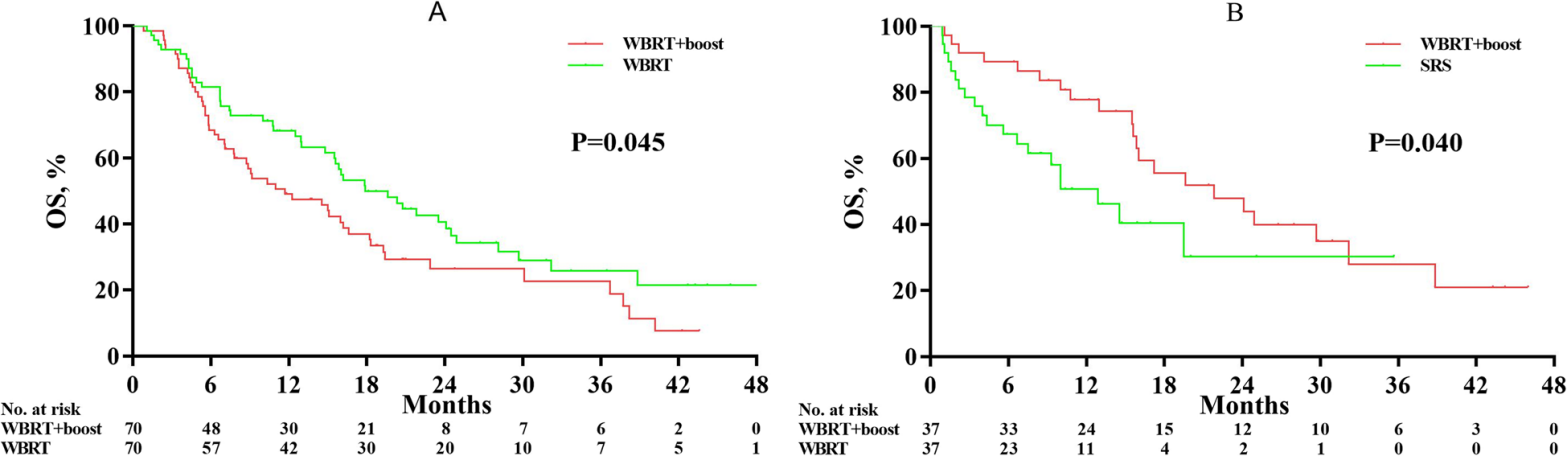
Overall survival analyses according to treatment group in matched patients after propensity score matching. The plots showed significant survival benefit of whole brain radiation therapy plus focal radiation boost (WBRT+boost) in 140 matched patients, compared to whole-brain radiation therapy (WBRT) alone (17.9 vs 11.7 months; *P* = 0.045; Fig. 3a). WBRT+boost group also resulted in significant survival advantage over stereotactic radiosurgery (SRS) groups among 140 matched patients (21.8 vs 12.9 months; *P* = 0.040; Fig. 3b)

Optimal propensity score matching was used to further compare the survival time between WBRT+boost and WBRT or SRS alone groups to balance the confounding. The general characteristics of the patients between the two groups were balanced after propensity score matching (Tables 2, and 3). Patients who received WBRT+boost also led to significantly longer survival than did WBRT among 140 matched patients between WBRT+boost and WBRT alone groups (17.9 vs 11.7 months; Fig. 3a). Among 74 matched patients between WBRT+boost and SRS alone groups, patients who treated with WBRT+boost also led to a longer survival than did SRS alone (21.8 vs 12.9 months; *P* = 0.040; Fig. 3b).

**Table 2:** Baseline characteristics of patients between WBRT+boost and WBRT alone after propensity score matching

	WBRT + boost	WBRT Alone	*P*-Value
	(*n* = 70)	(*n* = 70)	
Age, No. (%)			0.611
< 60	34	31	
≥60	36	39	
Sex, No. (%)			0.577
Male	48	51	
Female	22	19	
KPS, No. (%)			0.294
≤80	41	47	
> 80	29	23	
Number of BMs, No. (%)			0.175
1–3	42	34	
> 3	28	36	
Maximum size of BM, No. (%)			0.176
< 20 mm	33	41	
≧20 mm	37	29	
Extracranial metastases, No. (%)			0.092
Yes	16	21	
None	12	4	
Unknown	42	45	
Symptoms of BM, No. (%)			0.735
Yes	35	33	
None	35	37	
Systematic treatment			0.237
CTx	50	56	
No-CTx	20	14	

Abbreviations: *WBRT+boost* whole brain radiotherapy plus focal radiation boost, *WBRT* whole brain radiation therapy, *SRS* stereotactic radiosurgery, *KPS* Karnofsky Performance Status, *BM* brain metastasis, *CTx* chemotherapy, *No-CTx* no chemotherapy

**Table 3:** Baseline characteristics of patients between WBRT+boost and SRS alone after propensity score matching

	WBRT + boost	SRS Alone	*P*-Value
	(*n* = 37)	(*n* = 37)	
Age, No. (%)			1.00
< 60	14	14	
≥60	23	23	
Sex, No. (%)			0.407
Male	27	30	
Female	10	7	
KPS, No. (%)			0.815
≤80	20	21	
> 80	17	16	
Number of BMs, No. (%)			0.744
1–3	31	32	
> 3	6	5	
Maximum size of BM, No. (%)			0.642
< 20 mm	18	20	
≧20 mm	19	17	
Extracranial metastases, No. (%)			0.317
Yes	2	6	
None	6	6	
Unknown	29	25	
Symptoms of BM, No. (%)			0.809
Yes	14	13	
None	23	24	
Systematic treatment			0.802
CTx	26	25	
No-CTx	11	12	

Abbreviations: *WBRT+boost* whole brain radiotherapy plus focal radiation boost, *SRS* stereotactic radiosurgery, *KPS* Karnofsky Performance Status, *BM* brain metastasis, *CTx* chemotherapy, *No-CTx* no chemotherapy

### Analyses of intracranial progression-free survival time

**Fig. 4:**
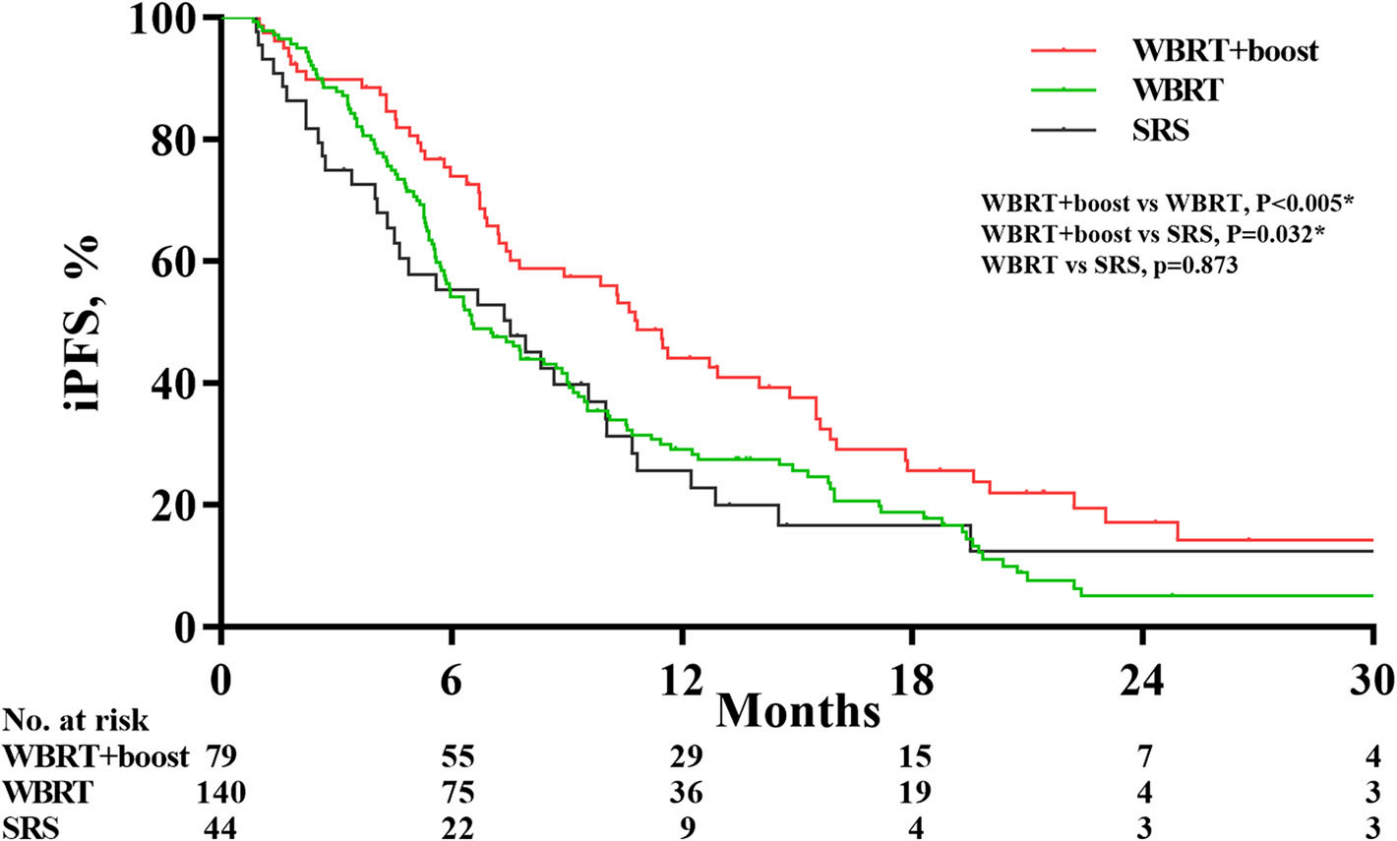
Intracranial progression-free survival analyses according to treatment group in all patients. Whole-brain radiation therapy plus focal radiation boost (WBRT+boost) group experienced a significantly longer intracranial progression-free survival than did whole brain radiation therapy (WBRT) alone (10.8 vs 6.5 months; *P* = 0.005) or stereotactic radiosurgery (SRS) alone (10.8 vs 7.5 months; *P* = 0.032)

The 1-year iPFS rates of the WBRT+boost group, WBRT group, and SRS group were 36.7, 25.7, and 20.5%, respectively. Similarly, there were significant differences in iPFS between WBRT+boost, WBRT, and SRS groups (10.8 vs 6.5 vs 7.5 months; *P* = 0.020). In pairwise comparison of the iPFS, WBRT+boost also had survival advantages over WBRT (10.8 vs 6.5 months; *P* = 0.005) and SRS (10.8 vs 7.5 months; *P* = 0.032) (Fig. 4).

### Univariate and multivariate analyses of overall survival

In univariate analyses, age, sex, number of brain metastases, extracranial metastases, systemic therapy, and radiotherapy were significant prognostic factors associated with OS. Multivariate analysis showed no or unknown extracranial metastases (HR, 0.63; HR, 0.61; *P* = 0.011), and female sex (HR, 0.58; *P* = 0.002) were related to increased survival. Multivariate analysis also showed patients with multiple brain metastases (> 3) (HR, 1.88; *P* < 0.001) or those who did not receive systemic chemotherapy (HR, 2.08; P < 0.001) had a poorer OS (Table 4).

**Table 4:** Univariate and multivariate analyses of factors influencing OS of lung cancer patients with brain metastases

Factors		Median OS, months	Univariate		Multivariate	
			HR (95% CI)	*P* Value	HR (95% CI)	*P* Value
Age, years	< 60	15.9		0.019		
	≥60	10.8	1.42 (1.06–1.91)			
Sex	Male	10.0		0.016		**0.002**
	Female	17.2	0.67 (0.48–0.93)		0.58 (0.41–0.81)	
KPS	≤80	11.0		0.059		
	> 80	14.8	0.75 (0.56–1.01)			
Number of BMs	1–3	19.5		< 0.001		**< 0.001**
	> 3	8.9	2.00 (1.48–2.71)		1.88 (1.36–2.59)	
Maximum size of	< 20	11.7		0.173		
BM, mm	≥20	12.9	1.22 (0.91–1.63)			
Symptoms of BM	Yes	12.3		0.198		
	None	12.9	0.83 (0.62–1.11)			
Extracranial metastases	Yes	7.5		0.003		**0.011**
	None	18.3	0.68 (0.43–1.06)		0.63 (0.40–0.99)	
	Unknown	15.0	0.57 (0.42–0.79)		0.61 (0.43–0.85)	
Systematic Tx	CTx	14.9		< 0.001		**< 0.001**
	No-CTx	6.0	1.83 (1.35–2.49)		2.08 (1.53–2.84)	
Radiotherapy	WBRT+boost	17.9		0.002		
	WBRT	8.7	1.82 (1.30–2.55)			
	SRS	14.5	1.23 (0.75–2.02)			

Abbreviations: *OS* overall survival, *HR* hazard ratio, *CI* confidence interval, *KPS* Karnofsky Performance Status, *BM* brain metastasis, *CTx* chemotherapy, *No-CTx* no chemotherapy, *WBRT* whole brain radiation therapy, *SRS* stereotactic radiosurgery, *WBRT+boost* whole brain radiotherapy plus focal radiation boost

## Discussion

This multi-center retrospective study evaluated different radiotherapy treatments for brain metastases in SCLC patients. Compared to WBRT alone, WBRT+boost was associated with superior OS and iPFS both in the entire cohort (*n* = 263) and matched cohort (*n* = 140). Patients who treated with WBRT+boost also experienced a longer OS than did SRS in matched cohort (*n* = 74).

Due to the properties of multiple metastases, WBRT was considered the standard treatment for brain metastases from SCLC patients. However, the optimal treatment for brain metastases remained controversial. Some published studies suggested that WBRT plus radiation boost was more suitable for the treatment of brain metastases in SCLC patients than WBRT alone. Sun et al. reported that, compared with WBRT alone, a longer survival was observed in patients who received WBRT plus radiation boost than did WBRT alone (13.4 vs 8.5 months; *p* = 0.004). To minimize the difference in the number of BMs between the two groups, a subgroup analysis was performed. Among patients with 1–3 brain metastases, WBRT plus radiation boost was also associated with longer OS than WBRT alone (13.4 vs 9.6 months; *p* = 0.022) [14]. Their findings were similar to ours. Wegner et al. reported that the longer OS was observed in SCLC patients who received WBRT plus SRS than did SRS alone (14 vs 6 months, *p* = 0.040). Of note, only 6 patients received WBRT plus SRS in their study [21]. Andrews et al. found that WBRT plus SRS group showed an improved KPS score and better OS than WBRT alone group in patients with single brain metastasis (6.5 vs 4.9 months; *P* = 0.0393). They also found that better control rates at 1 year in the WBRT plus SRS group (82%) vs 71%, *P* = 0.01). Of note, only 24 (7.2%) SCLC patients were included in their study [16].

Some studies suggested that SRS alone may be appropriate treatment for brain metastases in SCLC patients [13, 22]. Robin et al. reported SCLC patients who treated with upfront SRS was associated with longer OS than did WBRT ± SRS (10.8 vs 7.1 months, *p* < 0.001). However, in the subgroup analysis, there was no survival difference between SRS alone and WBRT plus SRS (*p* = 0.601) [22]. Bernhardt et al. also conducted a randomized controlled trial to investigate the treatment response of WBRT or SRS alone in SCLC patients. However, the final conclusions have not yet been published [13]. In a randomized controlled clinical trial published in JAMA, Brown et al. reported that WBRT plus SRS group had no survival difference compared with SRS alone group in patients with 1–3 brain metastases (7.4 vs 10.4 months; *P* = 0.92). However, the higher intracranial tumor control rates at 3 months was observed in patients who treated with WBRT plus SRS (93.7% vs 75.3%, *P* < 0.001) [11]. Of note, only 88 (66.7%) patients were lung cancer in their study. Therefore, their findings may not apply to SCLC patients. Aoyama et al. enrolled 132 patients with 1–4 brain metastases, among them, only 88 (66.7%) were lung cancer. They found that patients who treated with WBRT plus SRS group did not improve the survival time than did SRS alone (7.5 vs 8.0 months; *P* = 0.42), but significantly avoided the risk of intracranial recurrence [23]. Although previous studies were consistent with our findings, most of the above studies did not enroll SCLC patients alone. Combined with the characteristics of multiple metastases from SCLC and the better local control rates of WBRT, adjuvant WBRT combined with focal boost was necessary and indispensable for brain metastases from SCLC.

Our data also showed that the 1–3 brain metastases, and without extracranial metastases were prognostic factor for increased survival, which was similar to previous findings [24, 25]. We also found that gender and systemic treatment were prognostic factors for OS.

Our study also has several limitations. Firstly, the general characteristics of patients are not balanced. Of course, we performed propensity score matching to control the confounding between the WBRT+boost and WBRT or SRS groups. Secondly, RT-related neurotoxicity was not evaluated. Thirdly, the inherent characteristics of retrospective research and patient heterogeneity may bias the results.

## Conclusions

Among SCLC patients, the use of WBRT+boost resulted in both longer iPFS and OS than did WBRT or SRS alone. Due to the SCLC-derived intracranial dissemination and better intracranial control, WBRT+boost may be a preferred strategy for brain metastases in SCLC patients.

## Data Availability

The datasets used and/or analysed during the current study are available from the corresponding author on reasonable request.

## Abbreviations

3D-CRT: 3D conformal radiotherapy
CTV: Clinical target volume
GTV: Gross tumor volume
IMRT: Intensity-modulated radiation therapy
iPFS: Intracranial progression-free survival time.
OS: Overall survival
PCI: Prophylactic cranial irradiation
PTV: Planning tumor volume
SCLC: Small cell lung cancer
SRS: Stereotactic radiosurgery
WBRT: Whole brain radiotherapy
WBRT+boost: WBRT plus focal radiation boost
